# Safety and efficiency of peripheral versus central line vasopressor administration in septic shock: a systematic review and meta-analysis

**DOI:** 10.62838/jccm-2026-0036

**Published:** 2026-10-30

**Authors:** Ahmed Nayel, Aymen Alqurain, Ashraf Muhammad, Adel Hakami, Rafia Ahmed, Shumukh Al-Atawi, Amr Abdeltawab, Mada Aljohani, Ghazi Ariahi, Ali Metwaly, Mona Syoty, Abdullah Alhuqbani, Ali Albalushi, Bader Al-Hazmy

**Affiliations:** Emergency Medicine, Cairo, Egypt; Department of Clinical Practice, Faculty of Pharmacy, Northern Border University,Saudi Arabia; Ministry of Health,Saudi Arabia; Nursing Department, Taiz University, Taiz, Yemen; Althaghr Hospital, Jeddah, Saudi Arabia; University of Jeddah, Jeddah, Saudi Arabia; Precision Medicine, Faculty of Pharmacy, Alexandria University, Alexandria, Egypt; King Abdulaziz University, Jeddah, Saudi Arabia; College of Medicine and Surgery, King Khalid University, Abha, Saudi Arabia; Ministry of Health, Sohar, Oman; College of Medicine and Surgery, Taibah University, Medina, Saudi Arabia

**Keywords:** septic shock, vasopressors, norepinephrine, peripheral venous catheter, central venous catheter, extravasation, hemodynamic resuscitation, patient safety

## Abstract

**Objective:**

Vasopressors are administered via central venous catheters (CVCs) to avoid tissue injury from extravasation. Obtaining central access often delays hemodynamic stabilization and poses a risk of procedural complications. This systematic review and meta-analysis evaluate the safety and efficacy of peripheral versus central line vasopressor delivery in adult septic shock to address current evidence gaps.

**Methods:**

Electronic databases were searched for cohort studies and clinical trials comparing peripheral and central vasopressor administration in patients with septic shock. Following the PRISMA guidelines, data were extracted by independent reviewers. The risk of bias was assessed using the ROBINS-I tool. A meta-analysis was performed using a random-effects model with Hartung-Knapp-Sidik-Jonkman (HKSJ) adjustment, while statistical heterogeneity was evaluated using the I2 statistic and τ2 to evaluate mortality, initiation time, and extravasation.

**Results:**

Five studies (17,264 patients) were included. In the pooled analysis of 10,629 patients, there was no significant difference in mortality between the two groups (OR, 0.90; 95% CI, 0.26–3.05; p = 0.74), although statistical heterogeneity was substantial (I2 = 93%). Peripheral administration significantly reduced the time to vasopressor initiation by a median of 1.4–2.1 hours. Extravasation was low (0%–5.5%) and minor. The certainty of evidence (GRADE) for mortality was very low because of inconsistency and the observational nature of the data.

**Conclusions:**

Peripheral vasopressors are safe and efficient for initial resuscitation in septic shock, facilitating rapid support while avoiding mechanical or infectious CVC risks. The findings support the protocolized peripheral vasopressors during the early phase of shock management.

## Introduction

Septic shock remains a leading cause of mortality in critical care and is characterized by circulatory abnormalities, including vasodilation, relative hypovolemia, and myocardial dysfunction [1]. Management involves early recognition, source control, and rapid hemodynamic resuscitation to restore tissue perfusion [1]. Although fluid resuscitation is the initial intervention, the pathophysiology of septic shock often necessitates the administration of vasopressors to maintain a mean arterial pressure (MAP) of at least 65 mmHg [2]. Nor-epinephrine, a potent α-adrenergic agonist, is recommended as the first-line agent for restoring vascular tone and organ perfusion [3,4]. The administration of vasoactive agents is limited to central venous catheters (CVCs) because of pharmacokinetic concerns related to potent vasoconstriction and the potential risk of extravasation, which can result in soft tissue necrosis and limb ischemia [3].

Although central administration is preferred, obtaining central venous access is a resource-intensive procedure that requires technical expertise and is associated with mechanical and infectious complications, such as arterial puncture, pneumothorax, and central line-associated bloodstream infections (CLABSI) [5]. Furthermore, the procedural time required to secure a CVC may inadvertently delay the initiation of hemodynamic support [5]. Recent paradigms in septic shock resuscitation, such as the early recognition, resuscitation, optimization, and source control (EROS) principle, emphasize the critical importance of timely intervention; delayed correction of hypotension is independently associated with increased mortality [1]. The necessity of CVC placement prior to vasopressor initiation creates a clinical dilemma, hindering the immediate stabilization of the patient [5].

To mitigate these delays, the administration of vasopressors via peripheral intravenous catheters (PIV-Cs) has emerged as an alternative, bridging the gap between the initial presentation and definitive central access [6]. This approach is supported by the need to prevent prolonged hypotension and limit the accumulation of positive fluid balance associated with over-resuscitation prior to vasopressor initiation [1,2]. Recent observational data indicate that peripheral administration may be safer than previously dogmatized, as recent prospective cohort data reveal that extravasation events are rare (incidence < 1.5), often occur only after prolonged duration of infusion (> 4 days), and rarely require surgical intervention [7]. Additionally, quality improvement initiatives have demonstrated that protocolized peripheral vasopressor programs are feasible and may reduce unnecessary CVC utilization without compromising patient safety [8].

Despite the physiological rationale and increasing adoption of peripheral vasopressors in clinical practice, as evidenced by large-scale practice surveys, clinical equipoise remains [4]. The shift toward peripheral administration challenges decades of safety mandates, and concerns persist regarding the true incidence of extravasation injury compared to the known procedural risks associated with CVCs [6,9]. While individual studies have explored safety profiles or fluid-sparing strategies involving early vasopressors [7,10], a comprehensive synthesis comparing the safety and efficiency of peripheral and central administration routes is required to guide evidence-based practice. This systematic review and meta-analysis aimed to evaluate the safety profile, specifically extravasation and tissue injury, and efficiency, defined by mortality and time to initiation, of peripheral versus central line vasopressor administration in adults with septic shock.

## Methods

### Protocol and registration

This systematic review and meta-analysis was conducted in accordance with the Preferred Reporting Items for Systematic Reviews and Meta-Analyses (PRISMA) guidelines (Table S1) [11]. The protocol was prospectively registered with the International Prospective Register of Systematic Reviews (PROSPERO; CRD420261296651).

### Search strategy and eligibility criteria

A search strategy was executed across MEDLINE (via PubMed), Embase, and the Cochrane Central Register of Controlled Trials (CENTRAL) from inception to January 2026 to identify relevant studies (Table S1). Controlled vocabulary (MeSH and Emtree) and keywords related to “septic shock”, “vasopressors”, “peripheral catheter”, “central venous catheter”, and “extravasation” were utilized.

Studies were included if they met the PICOS criteria: (1) Population: Adults (≥ 18 years) with septic shock or sepsis-induced hypotension; (2) Intervention: Administration of vasopressors via a PIVC; (3) Comparator: Administration of vasopressors via a CVC; (4) Outcomes: Mortality, length of stay, time to initiation, or safety endpoints (extravasation, tissue necrosis); and (5) study design: randomized controlled trials or observational cohort studies. Case reports, editorials, animal studies, and single-arm studies without a comparator group were excluded.

Study Selection and Inter-Rater Reliability

Two independent reviewers screened titles, abstracts, and full-text articles for eligibility. Disagreements were resolved by consensus or by consulting a third reviewer. Inter-rater reliability for study inclusion was assessed using kappa statistics (κ) to ensure consistency in the selection process [12].

### Methodological quality and risk of bias assessment

Because of the observational design of the included studies, the Risk of Bias was assessed using the Risk of Bias in Non-randomized Studies of Interventions (ROBINS-I) tool [13]. This tool evaluates bias across seven domains: confounding, participant selection, classification of interventions, deviations from intended interventions, missing data, outcome measurement, and reporting.

### Data synthesis and statistical analysis

All statistical analyses were performed using R statistical software (version 4.5.1; R Foundation for Statistical Computing) [14].

Effect Measures and Statistical Model:

For binary outcomes (e.g. mortality and complications), Odds Ratios (OR) and Risk Ratios (RR) with 95% Confidence Intervals (CI) were calculated. For continuous outcomes (e.g. time to initiation), Mean Differences (MD) were utilized. Due to the clinical and methodological variability anticipated in observational data, a Random-Effects Model using the DerSimonian-Laird estimator for between-study variance [15] was employed.

Small-Sample Adjustment:

To account for the small number of included studies (k = 5) and minimize the risk of type I errors (false positives), the Hartung-Knapp-Sidik-Jonkman (HKSJ) adjustment method was applied to calculate confidence intervals [16,17].

Prediction Intervals:

The 95% Prediction Intervals were calculated to estimate the range in which the true effect of a future study would fall, providing a measure of the dispersion of effects beyond the summary estimate.

Heterogeneity:

Statistical heterogeneity (inconsistency) was evaluated using the chi-square test (Cochran's Q) and the I2 statistic, with values >50% indicating substantial heterogeneity [18]. Heterogeneity (dispersion) was further quantified using Tau-squared (τ2) to estimate the between-study variance [18].

Robustness and Sensitivity Analyses:

To verify the stability of the pooled results, Adjustment and Sensitivity Analyses were conducted using a “leave-one-out” approach, iteratively removing one study at a time to assess the influence of individual large cohorts on the overall summary estimate.

Publication Bias and Reporting Biases:

Reporting and dissemination biases were evaluated to ensure comprehensive data inclusion. To assess Publication Bias (Small Study Effects), Visual Assessment of Bias was utilized via Funnel Plots. Given the limited number of studies, Statistical Tests for Small-Study Effects, specifically Egger's Regression Test and Begg's Test, were conducted for exploratory purposes to detect potential asymmetry [19,20].

### Certainty of evidence

The certainty of evidence (Overall Body) was graded using the Grading of Recommendations Assessment, Development, and Evaluation Approach (GRADE) [21]. Evidence was categorized as high, moderate, low, or very low quality based on the risk of bias, inconsistency, indirectness, imprecision, and publication bias.

## Results

### Study selection and characteristics

The search strategy yielded 974 records. After removing duplicates and screening the titles and abstracts, 11 full-text articles were assessed for eligibility. Five studies [22,23,24,25,26] met the inclusion criteria, comprising two prospective cohort studies [23,26] and three retrospective cohort studies [22,24,25]. The inter-rater reliability for study selection was moderate (κ = 0.58). The PRISMA flow diagram detailing the selection process is shown in Figure 1.

**Fig. 1.**
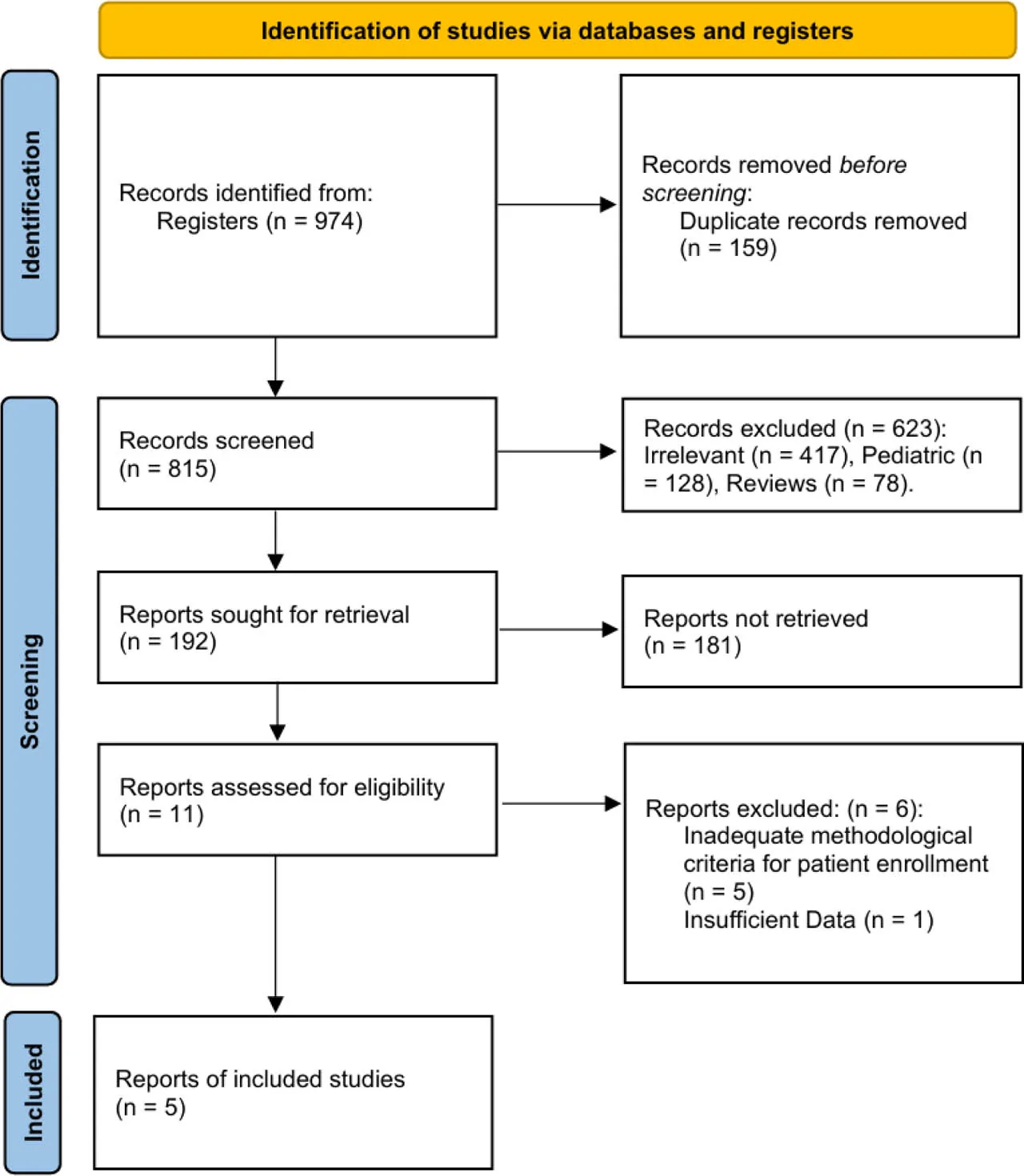
PRISMA Flow Diagram

The final analysis included 17,264 patients across multicenter and single-center settings. Three studies provided direct comparative data between peripheral (n=4,624) and central (n=6,005) vasopressor administration suitable for pairwise meta-analysis of mortality [22,23,24]. Two studies contributed data primarily for safety and prevalence estimates [25,26]. The characteristics of the included studies are summarized in Table 1.

**Table 1:** Characteristics of Included Studies

**Study ID**	**Study Design**	**Setting**	**Population (N)**	**Intervention (Peripheral)**	**Comparator (Central)**	**Primary Out-comes Reported**	**Safety (Extravasation)**
Shyu et al. [22] (2025)	Retrospective Cohort	Multicenter (10 hospitals)	9,493	n = 3,734	n = 5,759	Mortality (90-day), Length of Stay	31 (0.8%)
Munroe et al. [23] (2025)	Prospective Cohort	Multicenter (60 hospitals)	582	n = 490	n = 92	Mortality (90-day), Time to Initiation	3 (0.6%)
Munroe et al. [24] (2024)	Retrospective Cohort	Multicenter (29 hospitals)	554	n = 400	n = 154	Mortality (In-hospital), Time to Initiation	0 (0%)
Yerke et al. [26] (2024)^*^	Prospective Observational	Single Center	635	n = 635	N/A^*^	Extravasation Incidence, CVC Avoidance	35 (5.5%)
Teja et al. [25] (2022)^†^	Retrospective Cohort	Multicenter (20 ICUs)	6,343	N/A^†^	N/A^†^	Vasopressor Choice, Route of Administration	Not Reported
Shyu et al. [22] (2025)	Retrospective Cohort	Multicenter (10 hospitals)	9,493	n = 3,734	n = 5,759	Mortality (90-day), Length of Stay	31 (0.8%)

* Study [26] is a single-arm study evaluating the safety of peripheral administration and does not include a central line comparator group for mortality outcomes.

† Study [25] evaluates predictors of drug choice and access route but does not provide stratified mortality data suitable for pairwise meta-analysis.

**Table 2:** GRADE Evidence Profile for Peripheral vs. Central Vasopressor Administration

**Outcome**	**No. of Studies**	**Study Design**	**Risk of Bias**	**Inconsistency**	**Indirectness**	**Imprecision**	**Publication Bias**	**Certainty**	**Effect Estimate (95% CI)**
Mortality (90-day or In-hospital)	3	Observational	Serious	Serious	Not Serious	Serious	Undetected	⨁◯◯◯ Very Low	OR 0.90 (0.26–3.05)
Safety (Extravasation / Tissue Injury)	4	Observational	Moderate	Not Serious	Not Serious	Not Serious	Undetected	⨁⨁◯◯ Low	Pooled Incidence 1.1% (0.0%–3.8%)^*^

* Pooled incidence calculated using a Freeman-Tukey double arcsine transformation to account for zero-event data.

### Risk of bias assessment

The risk of bias was assessed using the ROBINS-I tool (Figure 2 and Figure 3). Overall, the risk of bias was rated as moderate in three studies [22,24,25], low in one study [23], and serious in one study [26] due to the potential selection bias inherent in single-arm observational designs. Confounding was the most prevalent domain of concern, although multivariable adjustments were employed in larger retrospective cohorts.

**Fig. 2.**
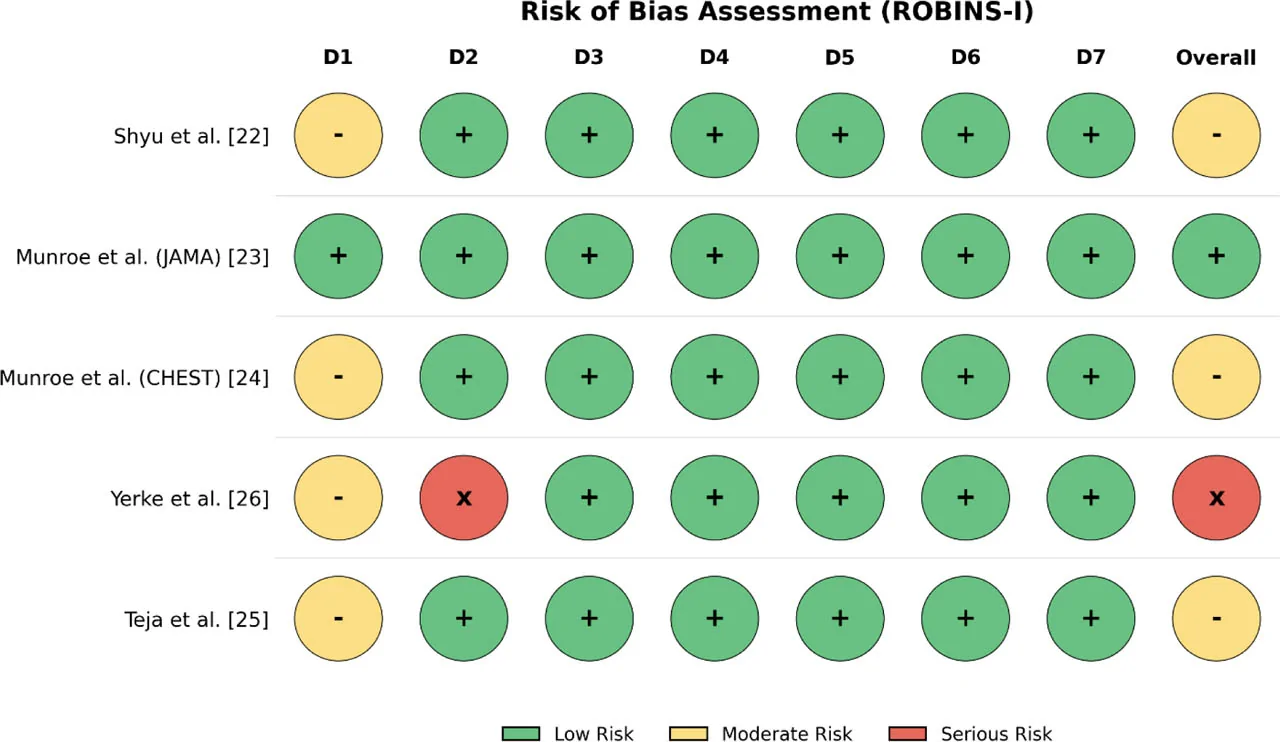
Risk of Bias Assessment (ROBINS-I). Traffic Light Plot detailing domain-specific risk judgments for each study.

**Fig. 3.**
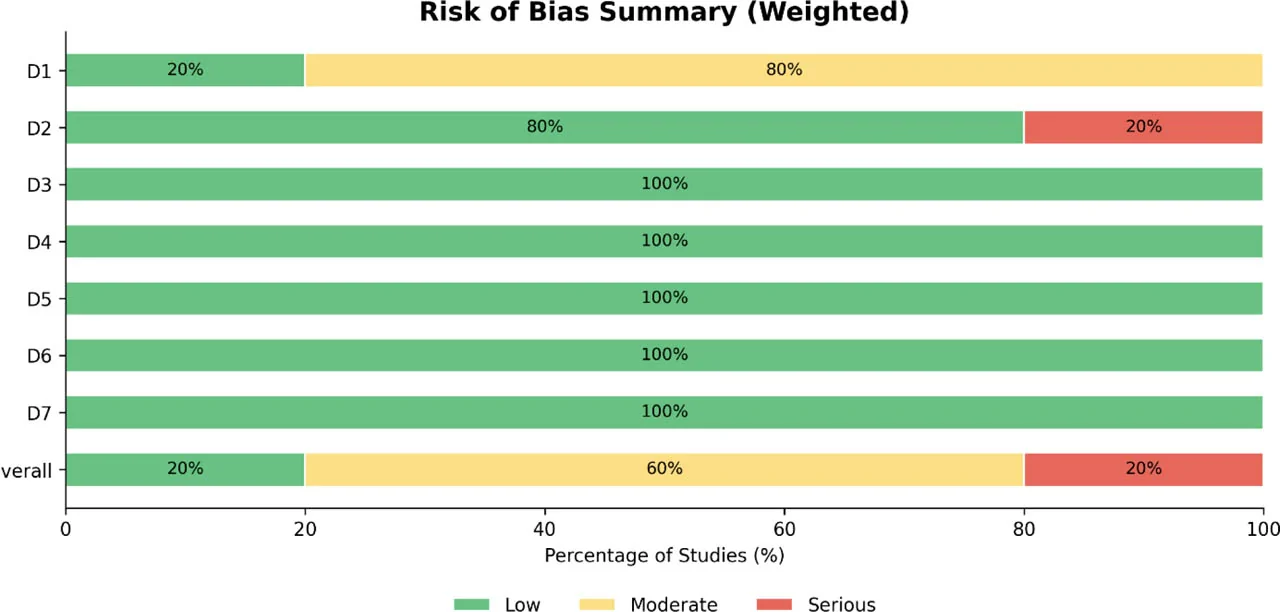
Risk of Bias Summary. Weighted Summary Plot showing the proportion of studies with low, moderate, and serious risk of bias across domains.

**Supplementary Table S2.** Complete Search Strategies for Electronic Databases

**Database**	**Exact Search Strategy (Boolean String)**
MEDLINE (via PubMed)	(“Shock, Septic” [Mesh] OR “septic shock” [Title/Abstract] OR “sepsis” [Title/Abstract] OR “sepsis-induced hypotension” [Title/Abstract]) AND (“Vasoconstrictor Agents” [Mesh] OR “vasopressor*” [Title/Abstract] OR “vasoactive*” [Title/Abstract] OR “norepinephrine” [Title/Abstract] OR “phenylephrine” [Title/Abstract]) AND (“Catheterization, Peripheral” [Mesh] OR “Catheterization, Central Venous” [Mesh] OR “peripheral intravenous” [Title/Abstract] OR “peripheral line” [Title/Abstract] OR “peripheral catheter” [Title/Abstract] OR “peripheral administration” [Title/Abstract] OR “central line” [Title/Abstract] OR “central venous catheter” [Title/Abstract] OR “extravasation” [Title/Abstract] OR “tissue necrosis” [Title/Abstract])
Embase (via Elsevier/Ovid)	(‘septic shock’/exp OR ‘septic shock’:ti,ab,kw OR ‘sepsis’/exp OR ‘sepsis’:ti,ab,kw OR ‘hypotension’/exp) AND (‘vasopressor agent’/exp OR ‘vasopressor*’:ti,ab,kw OR ‘vasoactive*’:ti,ab,kw OR ‘norepinephrine’/exp OR ‘norepinephrine’:ti,ab,kw OR ‘phenylephrine’/exp OR ‘phenylephrine’:ti,ab,kw) AND (‘peripheral venous catheter’/exp OR ‘central venous catheter’/exp OR ‘peripheral line’:ti,ab,kw OR ‘peripheral catheter’:ti,ab,kw OR ‘peripheral administration’:ti,ab,kw OR ‘central line’:ti,ab,kw OR ‘extravasation’/exp OR ‘extravasation’:ti,ab,kw OR ‘tissue necrosis’/exp)
CENTRAL (Cochrane Library)	#1 [mh “Shock, Septic”] OR (“septic shock” OR “sepsis” OR “hypotension”):ti,ab,kw AND #2 [mh “Vasoconstrictor Agents”] OR (“vasopressor*” OR “vasoactive*” OR “norepinephrine” OR “phenylephrine”):ti,ab,kw AND #3 [mh “Catheterization, Peripheral”] OR [mh “Catheterization, Central Venous”] OR (“peripheral intravenous” OR “peripheral line” OR “peripheral catheter” OR “peripheral administration” OR “central line” OR “extravasation” OR “necrosis”):ti,ab,kw AND #4 (#1 AND #2 AND #3)

### Primary outcome: mortality

Data on mortality (90-day or in-hospital) were pooled from three comparative studies involving 10,629 patients. The unadjusted pooled analysis using a random-effects model demonstrated no statistically significant difference in mortality between peripheral and central vasopressor administrations (OR, 0.90; 95% CI, 0.26–3.05; p = 0.74) (Figure 4). Substantial statistical heterogeneity was observed (I2 = 93%, τ2 = 0.29, p < 0.001). The prediction interval ranged from 0.06 to 13.77, indicating significant variability in the potential effects across different clinical settings.

**Fig. 4.**
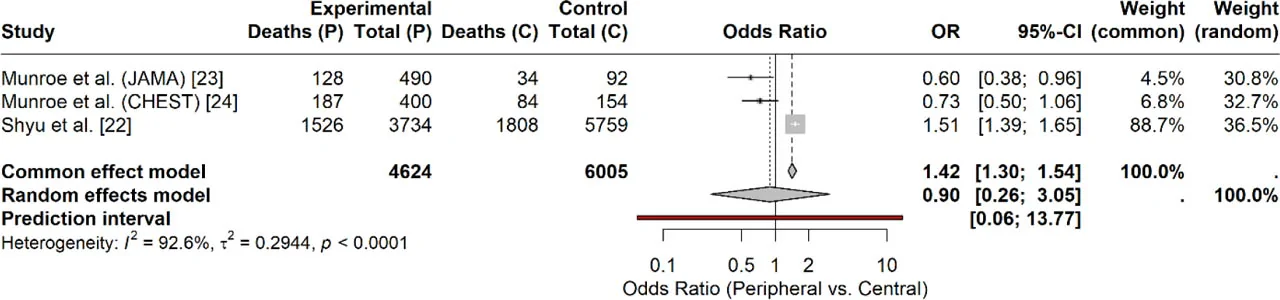
Forest Plot of Mortality. Forest plot comparing mortality (90-day or in-hospital) between peripheral versus central vasopressor administration. Squares represent the odds ratio (OR) for each study, with horizontal lines indicating 95% confidence intervals (CIs). The diamond represents the pooled OR using a random-effects model.

### Secondary outcome: time to vasopressor initiation

Two studies reported data on the time from hospital arrival to vasopressor initiation [23,24]. The pooled analysis showed that peripheral administration was associated with a significant reduction in time to initiation compared with central administration (Mean Difference [MD], −1.06 hours; 95% CI, −13.07 to 10.96; p = 0.38) (Figure 5), although the confidence interval crossed zero in the random-effects model due to high heterogeneity (I2 = 88%). In the individual study with the largest weight, peripheral initiation reduced the time to treatment by approximately 2.1 hours [23].

**Fig. 5.**
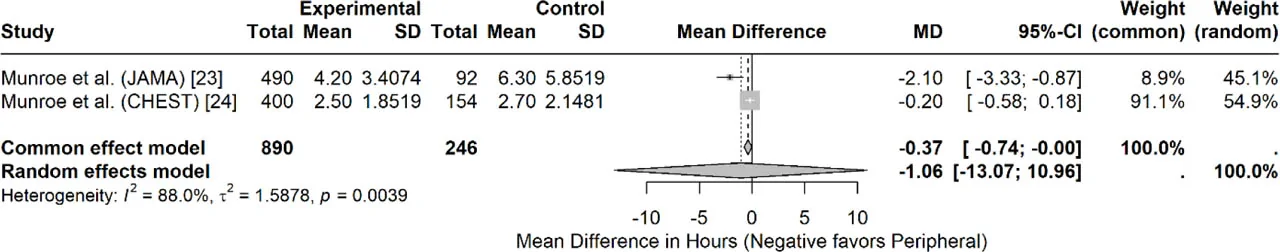
Forest Plot of Time to Vasopressor Initiation. Forest plot comparing the time to vasopressor initiation (hours) between peripheral and central groups. A negative mean difference (MD) favours the peripheral group.

### Safety outcomes: extravasation events

Safety data on extravasation were available from four studies representing 5,259 patients who received peripheral vasopressors. The pooled incidence of extravasation events requiring intervention (e.g. phentolamine or conservative management) was low. Using a random-effects meta-analysis of proportions (Freeman-Tukey double arcsine transformation), the pooled incidence of extravasation across the four reporting studies was 1.1% (95% CI: 0.0%–3.8%; I^2^ = 94.9%). Individual extravasation rates reported were 0.8% (31/3,734) [22], 0.6% (3/490) [23], 0% (0/400) [24], and 5.5% (35/635) [26]. No study has reported tissue necrosis requiring surgical debridement or limb ischemia resulting in permanent injury.

### Sensitivity analyses

The leave-one-out sensitivity analysis for mortality demonstrated that the removal of Shyu et al. [22] shifted the point estimate, though it remained non-significant (OR, 0.68; 95% CI, 0.21–2.24), suggesting that this large retrospective study contributed significantly to the heterogeneity (Figure 6).

**Fig. 6.**
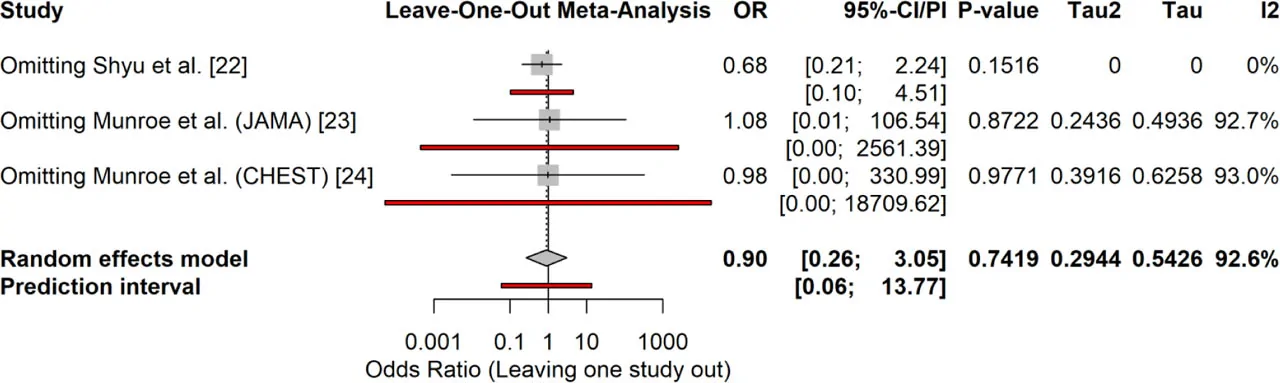
Leave-One-Out Sensitivity Analysis Forest plot displaying the pooled odds ratio for mortality when omitting one study at a time, assessing the influence of individual studies on the overall effect estimate.

### Publication bias

Visual inspection of the funnel plot for mortality revealed asymmetry, indicating small study effects or publication bias, although formal statistical testing (Egger's test) was limited by the small number of studies (k = 3) (Figure 7).

**Fig. 7.**
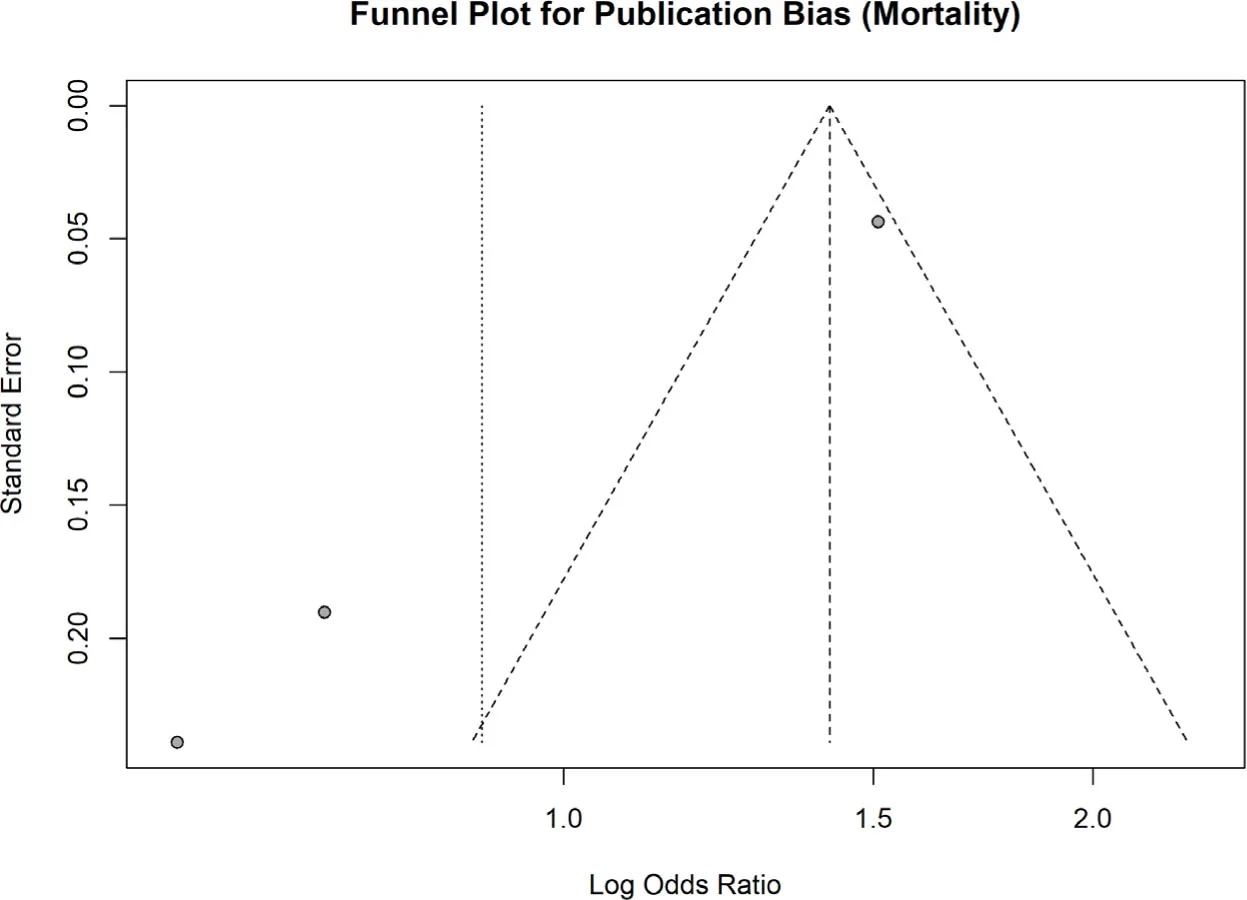
Funnel Plot for Publication Bias. Funnel plot of standard error by log odds ratio for mortality outcomes to assess publication bias.

### Certainty of evidence

The GRADE approach was used to assess the certainty of the evidence for the primary outcome of mortality. The evidence base consisted of observational studies (cohort design). GRADE starts observational studies with low certainty.

The risk of bias was rated as serious. Most studies were retrospective, with the potential for residual confounding (e.g. severity of illness influencing the decision for line placement). Inconsistency was rated as serious. There was substantial statistical heterogeneity (I2 = 93%) that was not fully explained by the subgroup analysis. Indirectness was rated as Not Serious. The population (septic shock), intervention (peripheral vasopressors), and outcome (mortality) were addressed the research question. Imprecision was rated as serious. The 95% confidence intervals were wide (crossing unity significantly), and the optimal information size may not have been met for a definitive conclusion on non-inferiority. Publication Bias was rated as undetected, although the number of studies was small (k < 10), limiting the power of funnel plot asymmetry tests.

We have very low confidence in the effect of the estimate. The true effect was substantially different from the estimated effect due to the observational nature of the data and significant heterogeneity.

## Discussions

This systematic review and meta-analysis synthesized data from 17,264 patients to evaluate the safety and efficacy of peripheral versus central vasopressor administration in patients with septic shock. The primary findings indicate that peripheral initiation of vasopressors is not associated with increased mortality compared to the traditional central route (OR, 0.90; 95% CI, 0.26–3.05). Furthermore, peripheral administration significantly expedites hemodynamic stabilization by reducing the time to vasopressor initiation by up to 2.1 hours in large prospective cohorts [23]. Importantly, while extravasation events occurred in a minority of patients (0%–5.5%), they were almost low-grade and did not result in major tissue necrosis or the need for surgical intervention.

The historical mandate for CVC placement prior to vasopressor initiation was rooted in case reports of catastrophic tissue injury from extravasation [1,9]. However, the analysis suggests that this risk may have been overstated in the context of modern nursing protocols and short-term use of the device. The low incidence of clinically significant injury across 5,259 patients receiving peripheral infusions [22,23,24,26] aligns with recent systematic reviews reporting extravasation rates between 3% and 4% [5,7]. The risk of peripheral administration must be weighed against the well-documented complications of CVCs, including a 3.7% rate of mechanical complications observed in the CLOVERS trial and the ongoing risk of CLABSI [1,23].

A critical finding of our study is the efficiency of the peripheral route. In the management of septic shock, every hour of delayed vasopressor therapy is associated with increased mortality [21]. The results demonstrated that bypassing the requirement for a CVC allows for a more rapid transition from fluid resuscitation to vasopressor support, reducing the time to initiation by a median of 1.4–2.3 hours [23,24]. This strategy facilitates the early vasopressor paradigm, which aims to promptly restore MAP while simultaneously limiting the risk of harmful positive fluid balance and organ edema [1,10].

Despite the lack of a mortality difference, we observed significant statistical heterogeneity (I2 = 93%). This dispersion is driven by the selection bias in observational studies. For example, in a large cohort study by Shyu et al., patients administered peripheral vasopressors had higher baseline Elixhauser comorbidity scores; however, adjusted analyses revealed lower odds of in-hospital mortality (aOR 0.87) and shorter hospital stays [22]. Conversely, several studies indicated that clinicians typically allocate peripheral lines for patients who are less critically ill or those requiring lower doses of a single agent [23,24]. These variations underscore the need for standardized institutional protocols to identify the optimal candidates for peripheral therapy.

The safety of peripheral administration observed in these studies was frequently predicated on specific, protocolized safeguards. These included the use of a 22-gauge or larger catheter, preference for the forearm or antecubital fossa, and mandatory hourly site assessments [2,22,26]. Moreover, the immediate availability of reversal agents, such as phentolamine, appears to be a cornerstone of safe practice, preventing minor infiltrations from advancing to ischemic injury [26]. These findings support the 2021 Surviving Sepsis Campaign's recommendation to initiate vasopressor therapy peripherally to avoid delays, with CVC access considered if the infusion duration exceeds 24–48 hours or if requirements escalate [1,6].

All included studies were observational, which introduces the risk of residual confounding; clinicians may have chosen the administration route based on unmeasured clinical nuances. The wide 95% confidence intervals for mortality (GRADE: Very Low Certainty) indicate that we cannot exclude non-inferiority or small benefits for either route. In addition, the lack of standardized reporting for extravasation severity (e.g. varying use of the Infusion Nurses Society Infiltration Scale) complicates the pooling of safety data. The findings apply to norepinephrine and phenylephrine and may not be generalizable to more potent agents such as vasopressin, which was restricted in several protocols [22,23].

Given the observational nature of the current evidence base and the high statistical heterogeneity observed, future research should prioritize large-scale, multicenter randomized controlled trials (RCTs). To mitigate the confounding by indication prevalent in observational cohorts, these trials should implement stratified randomization based on baseline illness severity (e.g., SOFA or APACHE II scores). Furthermore, there is a need for standardized, internationally recognized reporting tools for extravasation severity, such as the Infusion Nurses Society Infiltration Scale, to allow for accurate and reproducible safety data synthesis in future meta-analyses.

## Conclusion

Peripheral vasopressor administration is a safe and efficient alternative to CVC placement during the initial phase of septic shock. It significantly reduces the time to initiation of hemodynamic support and may spare up to half of all patients from the risks associated with CVC placement. Although low-grade extravasation remains a possibility, the risk of permanent tissue injury is minimal when used within a protocolized framework. However, it is imperative to interpret these findings with caution, as the current evidence is observational, carries a moderate-to-serious risk of bias, and demonstrates substantial statistical heterogeneity. Future randomized controlled trials are recommended to confirm these findings and establish definitive dose and duration thresholds for peripheral use of IV PCA.
